# Plant functional types do not predict biomass responses to removal and fertilization in Alaskan tussock tundra

**DOI:** 10.1111/j.1365-2745.2008.01378.x

**Published:** 2008-07

**Authors:** M Syndonia Bret-Harte, Michelle C Mack, Gregory R Goldsmith, Daniel B Sloan, Jennie DeMarco, Gaius R Shaver, Peter M Ray, Zy Biesinger, F Stuart Chapin

**Affiliations:** 1 Institute of Arctic Biology, University of AlaskaFairbanks, AK 99775, USA; 2 Department of Botany, University of FloridaGainesville, FL 32611, USA; 3 Department of Integrative Biology, University of CaliforniaBerkeley, CA 94720, USA; 4 The Ecosystems Center, Marine Biological LaboratoryWoods Hole, MA 05432, USA; 5 Department of Biology, Stanford UniversityStanford, CA 94305, USA

**Keywords:** arctic tundra, biodiversity, biomass compensation, nitrogen, plant functional types, productivity, species interactions, species removal, soil nutrient availability

## Abstract

1. Plant communities in natural ecosystems are changing and species are being lost due to anthropogenic impacts including global warming and increasing nitrogen (N) deposition. We removed dominant species, combinations of species and entire functional types from Alaskan tussock tundra, in the presence and absence of fertilization, to examine the effects of non-random species loss on plant interactions and ecosystem functioning.
2. After 6 years, growth of remaining species had compensated for biomass loss due to removal in all treatments except the combined removal of moss, *Betula nana* and *Ledum palustre* (MBL), which removed the most biomass. Total vascular plant production returned to control levels in all removal treatments, including MBL. Inorganic soil nutrient availability, as indexed by resins, returned to control levels in all unfertilized removal treatments, except MBL.
3. Although biomass compensation occurred, the species that provided most of the compensating biomass in any given treatment were not from the same functional type (growth form) as the removed species. This provides empirical evidence that functional types based on effect traits are not the same as functional types based on response to perturbation. Calculations based on redistributing N from the removed species to the remaining species suggested that dominant species from other functional types contributed most of the compensatory biomass.
4. Fertilization did not increase total plant community biomass, because increases in graminoid and deciduous shrub biomass were offset by decreases in evergreen shrub, moss and lichen biomass. Fertilization greatly increased inorganic soil nutrient availability.
5. In fertilized removal treatments, deciduous shrubs and graminoids grew more than expected based on their performance in the fertilized intact community, while evergreen shrubs, mosses and lichens all grew less than expected. Deciduous shrubs performed better than graminoids when *B. nana* was present, but not when it had been removed.
6. *Synthesis*. Terrestrial ecosystem response to warmer temperatures and greater nutrient availability in the Arctic may result in vegetative stable-states dominated by either deciduous shrubs or graminoids. The current relative abundance of these dominant growth forms may serve as a predictor for future vegetation composition.

## Introduction

Plant species differ in traits that affect carbon (C) and nitrogen (N) cycling, including litter quality, resource use strategy and feedbacks to disturbance regimes (Hobbie 1992; Craine *et al*. 2002; Chapin 2003; Ehrenfeld 2003). Thus, changes in plant abundance and diversity caused by anthropogenic activities can affect ecosystem function. Anthropogenic activities are changing plant community composition at an unprecedented scale and rate, both through direct effects on climate and element cycling, and by mediating the introduction and extinction of species (Vitousek *et al*. 1997; Sala *et al*. 2000; Chapin 2003). Rapid climate change, especially at high latitudes (McBean *et al*. 2005), and the global increase in the fixation and deposition of N, which often limits productivity of terrestrial ecosystems, are of particular importance to natural ecosystems (Vitousek *et al*. 1997; Galloway *et al*. 2004). Climate warming will further increase nutrient availability where low temperatures limit decomposition (Nadelhoffer *et al*. 1992; Giblin *et al*. 1994). Understanding how changes in plant species composition driven by these factors will affect ecosystem functioning is thus a high priority. Effects of changing species composition on ecosystem functioning will depend on both the traits of species that decline or disappear and the traits of species that replace them (Díaz *et al*. 2003; Suding *et al*. 2006).

Removal experiments offer a method to assess the effects of changing species composition on plant interactions and ecosystem functioning, especially in ecosystems dominated by long-lived, perennial species (Díaz *et al*. 2003). There is now considerable literature on the effects of experimental plant species removals in arctic and alpine systems, though few experiments have been maintained over the long-term (but see Aksenova *et al*. 1998). These experiments have sometimes been referred to as neighbour removal experiments, but because they might be confused with experiments involving non-specific removal of the nearest neighbours to target individuals, we will refer to them here simply as removal experiments. Predominantly positive responses of remaining plant biomass or cover to removal have been reported (del Moral 1983; Herben *et al*. 1997; Theodose & Bowman 1997; Aksenova *et al*. 1998; Gerdol *et al*. 2000; Gerdol *et al*. 2002). Negative responses have also been observed for different species in the same experiment, or under different environmental conditions (Shevtsova *et al*. 1995; Shevtsova *et al*. 1997; Aksenova *et al*. 1998; Wipf *et al*. 2006). Some removal experiments in arctic tundra have reported few plant responses, either positive or negative (Jonasson 1992; Hobbie *et al*. 1999; Bret-Harte *et al*. 2004). Facilitation should be more common in stressful or low productivity environments, and competition more prevalent in high productivity environments (Bertness & Callaway 1994; Brooker & Callaghan 1998). This hypothesis is supported by removal experiments and observational studies along alpine gradients (Choler *et al*. 2001; Callaway *et al*. 2002; Totland *et al*. 2004). While it is not surprising that positive, negative, and neutral interactions should all be seen among members of the plant community, the trajectory of ecosystem response following species loss and the long-term effects on ecosystem functioning will depend on the characteristics of the remaining plant species that respond most positively to the loss.

Complete biomass compensation is considered to have occurred if the growth response of the remaining plant species is sufficient to bring total plant biomass to pre-removal levels. Compensation may be similarly defined in terms of variables such as net primary production or N content of the vegetation. Biomass compensation by the remaining plants depends on reproductive output, recruitment, and vegetative growth. The last factor is especially important in the Arctic where the vast majority of plants are long-lived, clonal perennials.

Since 1997, we have been investigating how species traits and diversity affect ecosystem processing of C and N in arctic tussock tundra at Toolik Lake, Alaska, by means of a field experiment removing different combinations of species and entire plant functional types, with or without fertilization. We wished to understand (i) to what extent plant interactions affect the trajectory of community and ecosystem response to an environmental perturbation (fertilization), and (ii) the extent to which ecosystem capacity to respond to perturbation depends on the characteristics of individual species vs. those of plant functional types.

After considering plant traits that affect rates of nutrient cycling and C storage, such as stature, litter decomposability and thermal insulation, Chapin *et al*. (1996) concluded that species in a given physiognomic growth form (deciduous shrubs, evergreen shrubs, graminoids, forbs, mosses and lichens) are similar in their effects on ecosystem processes in tussock tundra, so that growth forms may be regarded as functional types. We designed our experiment based on this classification. In this study, we report the medium-term results (after 6 years) on biomass and N content of the remaining plant community following removal of the single dominant species from different functional types (evergreen shrubs, deciduous shrubs), all members of a single functional type (mosses), or members of three functional types (evergreen shrubs, deciduous shrubs, mosses). We did not manipulate graminoids because the dominant, *Eriophorum vaginatum*, is a tussock-forming sedge upon which many other species grow. Removing tussocks would have created a large disturbance and changed the microtopography and drainage.

In the short-term (after 2 years), we found that the remaining plants did not grow much in response to species or functional type loss, although growth was increased by fertilization (Bret-Harte *et al*. 2004). As a consequence of low compensatory growth, soil nutrient availability was greatly elevated by the removal treatments (Bret-Harte *et al*. 2004). These results suggested the following hypotheses. (i) Complete biomass compensation would occur eventually, because production in tussock-tundra is N-limited (Shaver & Chapin 1980, 1986; Chapin *et al*. 1995; Shaver *et al*. 2001) and removal greatly enhanced short-term nutrient availability. (ii) Biomass compensation would be due mostly to growth of remaining plants from the same functional type as the species removed, because species within a functional type use resources most similarly and should therefore demonstrate the strongest interspecific competition (Symstad 2003). (iii) Competitive interactions among plants should be most pronounced under fertilization, because alleviation of nutrient limitation may lead to light limitation.

We report here on the distribution of plant biomass and plant tissue N after 6 years of removal and fertilization.

## Methods

### site description and experimental treatments

The experiment was carried out in moist, acidic tussock tundra (Bliss & Matveyeva 1992) near Toolik Lake at the Arctic Long Term Ecological Research (LTER) site in the northern foothills of the Brooks Range, Alaska (68°38′ N, 149°34′ W, elevation 760 m). This vegetation contains approximately equal biomasses of graminoids (primarily *E. vaginatum* and *Carex bigelowii*), deciduous shrubs (*Betula nana*, with some *Vaccinium uliginosum* and *Salix pulchra*), evergreen shrubs (mainly *L. palustre* ssp. *decumbens* and *V. vitis-idaea*), and mosses (*Hylocomium splendens*, *Aulacomnium turgidum, Dicranum* spp. *Sphagnum* spp., etc.) (Shaver & Chapin 1991). Nomenclature follows Hultén (1968).

**Fig. 1:**
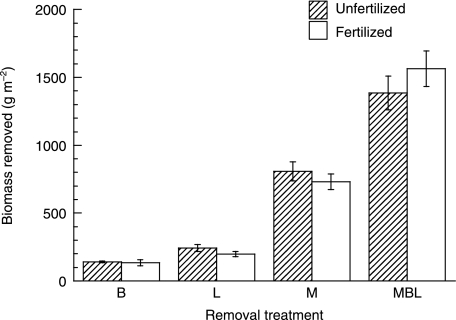
Total cumulative biomass removed from fertilized and unfertilized removal treatments between 1997 and 2003. Removal treatment abbreviation: B = removal of *Betula nana*, L = removal of *Ledum palustre*, M = removal of all mosses, MBL = combined removal of *B. nana*, *L. palustre*, and all mosses. Error bars indicate 1 SE among blocks (*n* = 6).

In 1997, we established six replicate blocks of 2 × 3 m plots separated from one another by buffer strips (1 or 2 m wide), in relatively uniform tussock tundra on a gentle (5%) north-facing slope, approximately 100 m south of the LTER experimental plots (Bret-Harte *et al*. 2001, Bret-Harte *et al*. 2002), as described in Bret-Harte *et al*. (2004). To avoid trampling, the plots were accessed from elevated boardwalks constructed in the buffer zones. From four randomly chosen plots within each block, one of the following species or combinations of species were removed, by methods described by Bret-Harte *et al*. (2004): *B. nana* (treatment B: dominant deciduous shrub); *L. palustre* (L: dominant evergreen shrub); all moss species (M; dominant non-vascular plants); or *B. nana, L. palustre*, and all mosses together (MBL). An additional four plots were randomly assigned to receive the same species removal treatments specified above, plus N and P fertilizers (treatment code followed by F). From two additional plots in each block no plants were removed, but the ground and vegetation were subjected annually to a mild physical disturbance simulating effects of removal. One of these plots (F) also received fertilization, while the other (C: control) did not. The remaining plots were assigned to other removal treatments (Bret-Harte *et al*. 2004) that were not sampled in the 2003 harvest reported here, due to logistical constraints. This included an undisturbed control treatment that was not harvested in 2003 because previous measurements showed no significant differences between disturbed and undisturbed controls (Bret-Harte *et al*. 2004). Removal treatments were maintained by annually removing, in early June, any regrowth of target species. The removed biomass was dried for 72 h at 65 °C and weighed (Fig. 1).

Each year in early June, 10 g N m^−2^ year^−1^ (as granular NH_4_NO_3_) and 5 g P m^−2^ year^−1^ (as commercial granular superphosphate) was applied to the fertilized treatments, using the same fertilization rate and method as in previous work (e.g. Shaver & Chapin 1980; Chapin *et al*. 1995; Bret-Harte *et al*. 2001; Bret-Harte *et al*. 2004). These rates exceed the natural inputs of nutrients (nearly four times the annual N requirement and nearly 20 times the annual P requirement of the vascular plants; Shaver & Chapin 1991). Our intent was not to simulate a particular scenario of N deposition under climate change, but rather to see the effects of altered species composition in an ecosystem relieved of nutrient limitation.

### environmental monitoring

Starting in 1997, soil temperature was measured 5 cm below the moss or soil surface in inter-tussock areas in three plots from each treatment harvested here, using copper–constantan thermocouples (after Bret-Harte *et al*. 2004). Thaw depth measurements were made at four locations in each plot in mid-August of 1997, 1999 and 2002, and in early September 2003 by pushing a probe from the moss surface to the bottom of the thawed soil in inter-tussock areas. Meteorological measurements made continuously by the Arctic LTER (Bret-Harte *et al*. 2001) 100 m from our plots, over the period of this experiment, are available at <http://ecosystems.mbl.edu/arc/home.htm.

### biomass harvests of remaining species

In late July 2003, we harvested the biomass of all plant species within 20 × 20 cm quadrats located randomly within each plot, by the method of Shaver & Chapin (1991). We harvested three quadrats per plot from three of the six replicate blocks, but (due to time constraints) only two quadrats per plot from the others, except that the third quadrat was harvested from C and F plots from one additional block. The rhizome-containing soil layer was harvested by cutting around each quadrat boundary with a serrated bread knife. All above-ground live vascular plant biomass, and all live rhizomes and below-ground stems within the quadrat boundaries, were separated by individual species. Current-year's growth from meristems located within the quadrat was included in the sample even if that growth extended outside the quadrat. New growth from meristems located outside, but that extended into the quadrat, was not included in the sample. From older stems that crossed the boundary, the portion within the quadrat boundaries was included.

As described by Shaver & Chapin (1991), biomass produced in the current year by each vascular plant was separated into leaves, new above-ground stems, and inflorescences with their peduncles, except that new growth from rhizomes was not separated but included with the previous years’ growth. Older biomass was separated into below-ground stems and rhizomes, above-ground stems, and (for evergreens) old leaves. Below-ground and above-ground old stems were separated at the position of the first adventitious roots. All graminoid and forb leaves, including both blade and sheath, were considered new biomass. Vascular plant litter and attached dead biomass were saved, but not separated by species. These biomass harvest methods are consistent with previous and ongoing LTER studies at the site and thus allow direct comparisons (Shaver & Chapin 1991; Chapin *et al*. 1995; Shaver *et al*. 2001).

Lichens and mosses (green portions only) were retained but not separated into species or new and old growth. Green moss biomass has been estimated to consist of approximately 20% new growth in those species where old and new growth can be distinguished, but they are not the majority of moss species present in tussock tundra (Chapin *et al*. 1995). All plant samples were dried at 60 °C for 72 h and weighed.

We calculated above-ground net primary productivity (NPP) for vascular plants as the total of the current-year's primary growth (new growth samples mentioned above), plus stem secondary growth for the three largest shrub species (*S. pulchra, B. nana*, and *L. palustre*). Secondary growth was calculated from old stem biomass and the relative secondary growth rates of stems in fertilized and unfertilized plots, as determined in a previous study of the adjacent LTER plots (Bret-Harte *et al*. 2002). As we did not have reliable measures of their growth, NPP was not calculated for non-vascular plants.

### nitrogen content analyses

We pooled material of each tissue type and species from all quadrats from a given plot. Pooled samples were ground in a Wiley mill with a #40 screen and analysed for N content by a Fisons Instruments elemental autoanalyser (Los Angeles, CA).

### soil inorganic nitrogen availability

We measured the 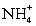 and 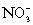 captured by mixed-bed ion exchange resins buried in the soil, to compare the relative availability of inorganic N in the different treatments (Giblin *et al*. 1991), by the method previously described (Bret-Harte *et al*. 2004), except that ions were extracted from the resins using KCl rather than NaCl/HCl. Three resin bags per plot (9 g FW resins each) were inserted about 3–5 cm below the surface in inter-tussock areas on 18–19 June 2003, and removed on 29 August 2003. Resin bags were washed free of soil using distilled water, then extracted in 100 mL 2 m KCl overnight. Extracts were frozen until analysis for 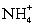 and 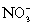 using an Astoria Pacific (Astoria, OR) colorimetric autoanalyser.

### statistical analyses

Biomass and production for each growth form were analysed by anova (GLM with block, removal treatment, fertilization, and a removal × fertilization interaction term; jmp Statistical Software). All data were tested for homogeneity of variance prior to analysis using Levene, Bartlett, O’Brien and Brown-Forsythe tests (JMP 2003). If two or more of the tests did not indicate homogeneity of variance, data were transformed with the algorithm *y* = 1n (*x* + 1) (Zar 1999). In some cases, due to high variability in the fertilized removal plots, data were inhomogeneous after transformation, and no other transformations (arcsine square root or 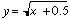 resulted in homogeneity. These data were ranked, and anova (same model as above) was conducted on the ranks (Zar 1999).

N contents of biomass and vascular plant production were calculated by multiplying the biomass of each tissue type of each species by the appropriate %N (from the pooled sample) for any given species tissue, and summing over all the species and tissues for each growth form in each quadrat. N content data were analysed using the statistical models given above.

Many species occurred so rarely that their biomass data could not be analysed separately, due in part to species turnover between fertilized and unfertilized treatments. Biomass was variable even for common species, because of the heterogeneity of vegetation at the scale of a 20 × 20 cm quadrat, and the statistical power to detect differences was low for the number of quadrats harvested. Post-experiment power analyses are not useful for interpreting non-significant results (Hoenig & Heisey 2001). Accordingly, we present statistical tests only for growth forms. All species present were included within the summed data for their growth form.

As an alternative approach to try to understand the response of remaining species, we calculated the expected biomass of species and growth forms within each treatment assuming that the remaining species took up N made available by removal in proportion to their N content in the intact community. We assumed that total N in above-ground biomass of the intact community was conserved in each removal treatment, and that there were no changes in N concentrations of plant tissues, or in allocation to different tissues within a species following removal. The N in the removed biomass was assigned to the remaining species in proportion to their N contents in the C treatment (for unfertilized removal treatments), or the F treatment (for fertilized removal treatments). Then we calculated expected biomass based on the new N mass of each species following the N redistribution (See Appendix S1 in Supplementary Material for details of the calculation). Because there were no changes to within-plant allocation, the assignment of N (and expected biomass) was basically proportional to the biomass of each remaining species in the control treatments. The difference between expected and observed biomass was expressed as a percentage of observed biomass.

## Results

### biomass removed

The total cumulative amount of biomass removed between 1997 and 2003 differed among treatments (Fig. 1). Removing the combination of all mosses, *B. nana* and *L. palustre* (MBL and MBLF) took out the most biomass, while removing *B. nana* alone (B and BF) took out the least, only 9.4% as much (Fig. 1). A large amount of moss was removed, in part because it was not practical to separate green and attached brown tissue in the field. Attached brown tissue was not included in our estimate of moss biomass in the harvest, in order to make it comparable to prior LTER harvests (e.g. Shaver & Chapin 1991), but was clearly greater than green moss biomass. Approximately 79–92% (depending on treatment) of the removed biomass was taken away in the first three seasons of the experiment (see Fig. S1). Regrowth by target vascular plants decreased rapidly as the experiment proceeded, but mosses re-colonized at a low level throughout the experiment (Fig. S1).

### remaining plant biomass

**Fig. 2:**
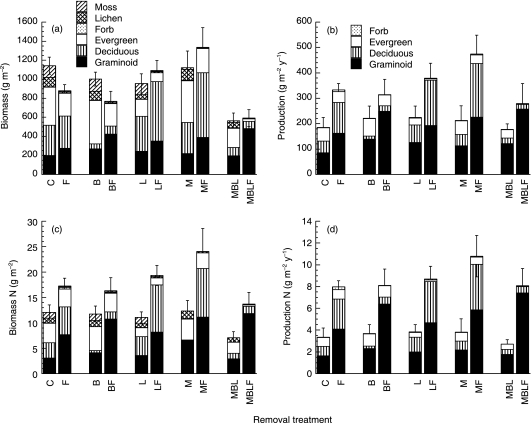
Biomass, production and N content of remaining plants by growth form (mosses, lichens, forbs, evergreen shrubs, deciduous shrubs and graminoids) in 2003, after 6 years of experimental removal and fertilization. (a) Biomass excluding roots, (b) above-ground net primary production of vascular plants, (c) mass of N in living biomass, (d) mass of N in above-ground net primary production of vascular plants. Treatment abbreviations: C = control (no removal, unfertilized), F = fertilized control (no removal), other abbreviations as in legend to Fig. 1. An F following a removal treatment abbreviation indicates that the treatment was fertilized. Error bars indicate 1 SE for the total community biomass, vascular plant production and N mass in production and biomass (all growth forms combined) among blocks (*n* = 6).

Total live biomass of remaining plants varied from 562 to 1335 g m^−2^ among the different removal and fertilization treatments (Fig. 2a). Evergreen shrubs had the most biomass of any growth form in control plots, but this was only slightly greater than that of deciduous shrubs or graminoids (Fig. 2a). Despite the visual prominence of *E. vaginatum*, graminoids had only slightly more biomass than green moss or lichens, and forbs were rare (Fig. 2a).

There was a significant effect of removal on total community biomass (Table 1). However, this was entirely because the two MBL removal treatments had less total biomass than controls (Fig. 2a, Table 1); the MBL treatment removed the most biomass. Total plant biomass did not differ significantly among the other removal treatments and controls, despite the cumulative removal of large amounts of biomass over the 6 years of the experiment (Fig. 1).

**Table 1:** Results of analysis of variance on live biomass of remaining plants by growth form and on accumulated litter, all treatments included in the analysis. *** *P* < 0.001, ** *P* < 0.01, * *P* < 0.05, † *P* < 0.1, ns non-significant (*P* > 0.1)

	Factor												
	
	Block			Removal (R)				Fertilization (Ft)			R × Ft		
				
Growth form	Ndf	Ddf	F	Ndf	Ddf	F	Post hoc	Ndf	Ddf	F	Ndf	Ddf	F
Deciduous shrubs‡	5	140	0.986 ns	4	140	34.401***	L > C; B,MBL < C	1	140	5.056* (↑; F > C)	4	140	1.696 ns
Evergreen shrubs‡	5	140	0.485 ns	4	140	14.724***	L,MBL < C,B,M	1	140	37.465*** (↓; F < C)	4	140	1.007 ns
Graminoids‡	5	140	0.947 ns	4	140	1.780 ns		1	140	8.531**↑	4	140	0.584 ns
Forbs§	5	140	0.318 ns	4	140	0.470 ns		1	140	0.035 ns	4	140	0.371 ns
Mosses§	5	140	1.748 ns	4	140	14.380***	M,MBL < C	1	140	217.05***↓	4	140	1.873 ns
Lichens§	5	140	0.475 ns	4	140	0.924 ns		1	140	195.68***↓	4	140	1.665 ns
Total live biomass‡	5	140	0.549 ns	4	140	12.447***	MBL < C	1	140	0.634 ns	4	140	2.133†
Total litter§	5	140	0.631 ns	4	140	2.063†		1	140	20.811***↑	4	140	0.018 ns

§ Data were log-transformed to achieve homogeneity of variance.

‡ Data were rank-transformed to achieve homogeneity of variance.

Fertilization did not significantly change total community biomass (Table 1), because some growth forms and species benefited at the expense of others (Fig. 2a). Under fertilization, deciduous shrubs and graminoids increased in treatments from which they were not removed, while evergreen shrubs, mosses and lichens all declined (Fig. 2a). Non-vascular plants virtually disappeared from most of the fertilized treatments.

Among the different growth forms, biomass of forbs and lichens showed no significant response to removal (Table 1). Green moss biomass was reduced by moss removal, and was not increased by any other treatment. Removal of either *L. palustre* or *B. nana*, the dominant species in their respective growth forms, caused significant reductions in their growth form biomass that were not completely compensated by the growth of other species within their growth forms (Fig. 2a, Table 1). However, some removal treatments increased the biomass of other growth forms. Removal of *L. palustre* significantly increased the biomass of deciduous shrubs (Table 1). Graminoid biomass was greatest under MBL removal, followed by *B. nana* removal, *L. palustre* removal, moss removal, and finally the control, even though the differences were not significant. Thus, graminoid biomass became greatest where deciduous shrub biomass was least, and least where all other components of the community were present.

### vascular plant production

Removal did not significantly affect total above-ground vascular plant production, indicating that complete compensation in vascular plant production had occurred by 2003 (Fig. 2b, Table 2). In contrast, total above-ground vascular plant production was greatly increased by fertilization (Fig. 2b, Table 2).

**Table 2:** Results of analysis of variance on above-ground net primary production of vascular plants, all treatments included in the analysis. *** *P* < 0.001, ** *P* < 0.01, * *P* < 0.05, † *P* < 0.1, ns non-significant (*P* > 0.1)

	Factor												
	
	Block			Removal (R)				Fertilization (Ft)			R × Ft		
				
Growth form	Ndf	Ddf	F	Ndf	Ddf	F	Post hoc	Ndf	Ddf	F	Ndf	Ddf	F
Deciduous shrubs‡	5	140	2.152†	4	140	32.331***	MBL,B < C,L,M	1	140	20.752*** (↑; F > C)	4	140	3.390*
Evergreen shrubs§	5	140	1.136 ns	4	140	21.505***	MBL,L < B,L,M	1	140	57.631*** (↓; F < C)	4	140	6.377***
Graminoids‡	5	140	0.684 ns	4	140	2.352†	C < MBL	1	140	13.494***↑	4	140	0.916 ns
Forbs§	5	140	0.391 ns	4	140	0.472 ns		1	140	0.278 ns	4	140	0.467 ns
Total production§	5	140	0.870 ns	4	140	2.481 ns		1	140	33.189***↑	4	140	1.291 ns

§ Data were log-transformed to achieve homogeneity of variance.

‡ Data were rank-transformed to achieve homogeneity of variance.

As in the case of biomass, removal of the dominant shrubs *B. nana* or *L. palustre* significantly decreased production by their respective growth forms. Thus, other members of the same growth form did not compensate for these removals (Fig. 2b, Table 2). In addition, there was a marginally significant effect of removal to increase graminoid production, because graminoid production was significantly greater under MBL removal than in controls. Removal did not affect forb production. Fertilization strongly promoted graminoid and deciduous shrub production, while evergreen shrub production declined (Fig. 2b, Table 2). However, there was a significant interaction between removal and fertilization for deciduous shrubs and evergreen shrubs. This occurred because fertilization promoted deciduous shrub production more when *B. nana* was present than when it was removed, and decreased evergreen shrub production more when *L. palustre* was removed than when it was present (Table 2).

### nitrogen content in plant biomass

Removal did not affect the N concentration of any plant tissues, but there was a redistribution of N among species and growth forms in the plant community under both fertilization and removal, due to changes in plant biomass. Only the two MBL removal treatments had significantly lower total N in live biomass than controls. *Ledum palustre* removal promoted N accumulation in deciduous shrubs (Fig. 2c, Table 3).

**Table 3:** Results of analysis of variance on the total N content of live plant biomass by growth form, all treatments included in the analysis. *** *P* < 0.001, ** *P* < 0.01, * *P* < 0.05, † *P* < 0.1, ns non-significant (*P* > 0.1)

	Factor												
	
	Block			Removal (R)				Fertilization (Ft)			R × Ft		
				
Growth form	Ndf	Ddf	F	Ndf	Ddf	F	Post hoc	Ndf	Ddf	F	Ndf	Ddf	F
Deciduous shrubs‡	5	140	1.327 ns	4	140	32.383***	L > C, B,MBL < C,M	1	140	23.822*** (↑; F > C)	4	140	2.040†
Evergreen shrubs¶	5	140	0.726 ns	4	140	15.560***	L,MBL < C,M,B	1	140	15.085*** (↓; F < C)	4	140	2.243†
Graminoids‡	5	140	0.550 ns	4	140	1.543 ns		1	140	34.871**↑	4	140	0.838 ns
Forbs¶	5	140	0.985 ns	4	140	1.039 ns		1	140	3.385†↑	4	140	0.479 ns
Mosses‡§	5	84	0.701 ns	2	84	0.290 ns		1	84	70.364***↓	2	84	0.368 ns
Lichens‡	5	140	1.062 ns	4	140	0.629 ns		1	140	154.35***↓	4	140	1.542 ns
Total live biomass‡	5	140	0.743 ns	4	140	7.128***	MBL < C,M,B,L	1	140	34.480***↑	4	140	0.711 ns
Total litter¶	5	140	0.995 ns	4	140	2.028†		1	140	82.309***↑	4	140	0.138 ns

¶ Data were log-transformed to achieve homogeneity of variance.

‡ Data were rank-transformed to achieve homogeneity of variance.

§ Homogeneity of variance could not be achieved while including the M, MBL removal treatments, because values in those treatments were so close to zero; these treatments were clearly less than the others.

In contrast, fertilization increased N concentrations in the tissues of all plants. The total live biomass N pool was increased by fertilization (Fig. 2c, Table 3). Under fertilization, N accumulated in deciduous shrubs and graminoids, but decreased in evergreen shrubs, mosses and lichens (Fig. 2c, Table 3).

### nitrogen content in vascular plant production

As expected, removal did not affect the amount of N in total vascular plant production, because N concentration in plant tissues did not change, and because there was complete compensation in total vascular plant production. Removal effects on the amount of N in production of different growth forms were similar to those seen for biomass production (Tables 2, 4).

**Table 4:** Results of analysis of variance on the N content of above-ground net primary production of vascular plants. *** *P* < 0.001, ** *P* < 0.01, * *P* < 0.05, † *P* < 0.1, ns non-significant (*P* > 0.1)

	Factor												
	
	Block			Removal (R)				Fertilization (Ft)			R × Ft		
				
Growth form	Ndf	Ddf	F	Ndf	Ddf	F	Post hoc	Ndf	Ddf	F	Ndf	Ddf	F
Deciduous shrubs‡	5	140	2.012†	4	140	27.038***	MBL,B < C,L,M	1	140	34.547*** (↑; F > C)	4	140	2.611*
Evergreen shrubs§	5	140	1.136 ns	4	140	21.505***	MBL,L < B,L,M	1	140	57.631*** (↓; F < C)	4	140	6.377***
Graminoids‡	5	140	0.652 ns	4	140	2.335†	C < MBL	1	140	39.840***↑	4	140	1.278 ns
Forbs§	5	140	1.036 ns	4	140	1.227 ns		1	140	3.538†↑	4	140	1.071 ns
*Total production*§	5	140	0.882 ns	4	140	1.422 ns		1	140	93.853***↑	4	140	0.384 ns

§ Data were log-transformed to achieve homogeneity of variance.

‡ Data were rank-transformed to achieve homogeneity of variance.

Fertilization significantly increased the amount of N contained in total vascular plant production (Fig. 2d, Table 4). Fertilization strongly increased the amount of N in production by graminoids and deciduous shrubs, and marginally by forbs (Fig. 2d, Table 4). The amount of N in production by fertilized evergreen shrubs decreased significantly despite increased N concentrations in evergreen shrub tissues, because of large reductions in evergreen shrub biomass and production in all fertilized treatments (Fig 2d, Table 4).

### species composition

**Fig. 3:**
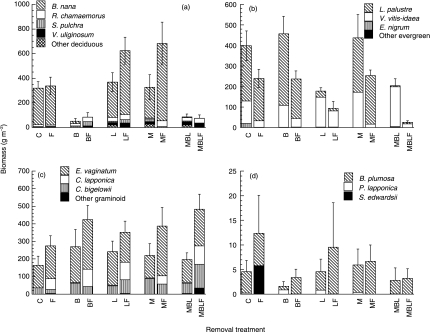
Biomass of the most common remaining vascular plant species within each growth form. Non-vascular plants were not separated to species. (a) Deciduous shrubs, (b) evergreen shrubs, (c) graminoids, (d) forbs. Abbreviations for removal treatments as in legend to Fig. 2. Full species names are as follows. Deciduous shrubs: *Betula nana, Rubus chamaemorus, Salix pulchra, Vaccinium uliginosum*, ‘other deciduous’ included *Arctostaphylos alpina* and *Salix phlebophylla*. Evergreen shrubs: *Ledum palustre, V. vitis-idaea, Empetrum nigrum*, ‘other evergreen’ included *Cassiope tetragona* and *Andromeda polifolia*. Graminoid: *Eriophorum vaginatum, Calamagrostis lapponica, Carex bigelowii*, ‘other graminoid’ included *Hierochloe alpina, Poa arctica, Luzula confusa, Luzula arctica* and *Eriophorum angustifolium*. Forbs: *Bistorta plumosa, Pedicularis lapponica, Stellaria edwardsii*, no other forbs were encountered. Error bars indicate 1 SE for the entire growth form (*n* = 6 blocks).

Most of the vascular growth forms included a single dominant species whose biomass accounted for much of the response of its growth form to the different treatments (Fig. 3). Most of the biomass response of deciduous shrubs was due to the dominant species, *B. nana*, in all treatments where it had not been removed (Fig. 3a). *Ledum palustre*, while not as dominant as *B. nana*, is the most abundant evergreen shrub and comprised more than half of the evergreen shrub biomass where it had not been removed (Fig. 3b). *Ledum palustre* declined under fertilization, but the subordinate evergreen *V. vitis-idaea* declined more. The dominant graminoid, *E. vaginatum* contributed much of the increased graminoid biomass in all fertilized treatments (Fig. 3c). However, *Calamagrostis lapponica*, a grass that was rare in control plots, responded strongly to fertilization, comprising between 12.1% and 28.5% of graminoid biomass in fertilized removal treatments (Fig. 3c). Forb biomass was largely comprised of *Bistorta plumosa*, except for abundant *Stellaria edwardsii* in one fertilized plot (Fig. 3d). We mistakenly identified *B. plumosa* as *B. bistortoides* in our previous paper (Bret-Harte *et al*. 2004).

### litter accumulation and n content

**Fig. 4:**
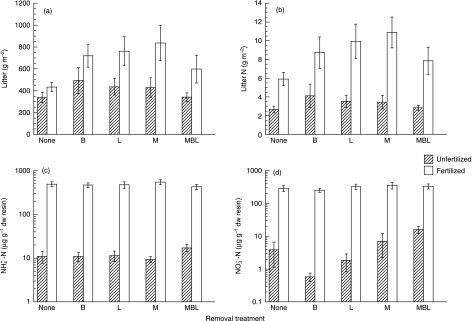
Above-ground litter (both loose and attached to living biomass) accumulated in removal treatments, and soil inorganic N availability in removal treatments, as measured by accumulation on ion exchange resins. (a) Mass of litter, (b) mass of N in litter, (c) the mass of N as 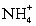, (d) the mass of N as 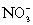. Note the logarithmic scale on the *y*-axis in panels (c) and (d). Abbreviations for removal treatments as in legend to Fig. 1. Error bars indicate 1 SE among blocks (*n* = 6).

In contrast to total community biomass, fertilization greatly enhanced the accumulation of above-ground litter (Fig. 4a, Table 1). There was a marginally significant fertilization by removal interaction because litter accumulation increased in some fertilized removal treatments more than others (Table 1). The most litter accumulated in fertilized removal treatments with the highest graminoid biomass (Fig. 4a), but *post hoc* tests were not significant.

The amount of N in litter was greatly increased by fertilization (Fig. 4b, Table 3). Litter N was nearly half of the N in total live biomass in some fertilized removal treatments. There was a trend toward higher litter N with removal (*P* = 0.088), again because graminoid-dominated removal treatments accumulated more litter than others (Table 3).

### nitrogen availability in soil, thaw depth and soil temperature

Availability of inorganic N in soil (both 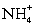 and 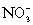), as measured by accumulation on ion exchange resins, was very low in unfertilized control plots (Fig. 4c,d). Removal did not affect available 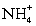, which varied little. Removal significantly increased available 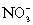 in soil (Fig. 4d, Table 5), because MBL plots had higher 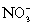 availability than control plots. Although there was an order of magnitude difference in available 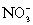 between the unfertilized removal treatments, 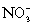 levels had been 2–3 orders of magnitude higher in unfertilized M and MBL plots earlier in the experiment (Bret-Harte *et al*. 2004). Inorganic soil nutrient availability had returned to more normal levels by 2003. Fertilization significantly increased availability of both 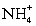 and 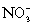 by 2–3 orders of magnitude (Fig. 4c,d, Table 5).

**Table 5:** Results of analysis of variance on inorganic N availability in soil and on depth of thaw. *** *P* < 0.001, ** *P* < 0.01, * *P* < 0.05, † *P* < 0.1, ns non-significant (*P* > 0.1)

	Factor												
	
	Block			Removal (R)				Fertilization (Ft)			R × Ft		
				
Variable	Ndf	Ddf	F	Ndf	Ddf	F	Post hoc	Ndf	Ddf	F	Ndf	Ddf	F
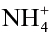 – N§	5	161	0.605 ns	4	161	0.299 ns		1	161	1090.67*** (↑; F > C)	4	161	1.082 ns
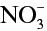 – N§	5	162	0.116 ns	4	162	6.651***	MBL > C,B,L,M	1	162	831.492*** (↑; F > C)	4	162	3.301 *
Thaw depth	5	249	2.970*	4	249	2.356†	C < MBL	1	249	21.112***↓	4	249	0.546 ns

§ Data were log-transformed to achieve homogeneity of variance.

When measured in August 2003, removal caused a marginally significant increase in depth of thaw, an integrated measure of soil temperature over the growing season, because thaw depth in both MBL treatments was an average of 2.4 cm (5.2%) greater than in controls (see Fig. S2, Table 5). In contrast, fertilization more significantly reduced depth of thaw, by an average of 8.5% across removal treatments (Fig. S2, Table 5). Despite this, neither fertilization nor removal significantly affected the sum of thawing degree-days at 5 cm depth (data not shown), perhaps due to high variance among the sensors in different plots.

### comparison of observed and expected biomass

We compared plant biomass observed in our unfertilized removal plots with the biomass expected if the N contained in the biomass of the removed species were redistributed to the remaining plants. This is a reasonable null hypothesis, because removal did not change tissue N concentrations of remaining species, and N limits growth in unfertilized tussock tundra (Shaver & Chapin 1980, 1986; Chapin *et al*. 1995; Shaver *et al*. 2001). Expected values of total live plant biomass agreed reasonably well with observed values. Deviations from expectation ranged from 6% to 15% of observed values in the different unfertilized removal treatments (Table 6). Observed biomass of deciduous shrubs, graminoids, and evergreen shrubs was a little greater than expected biomass in all unfertilized removal treatments, while observed biomass of mosses and lichens was slightly less than expected. The observed values for the different growth forms never deviated from expected values by > 13%. The greatest difference between observed and expected values occurred in the unfertilized MBL treatment.

**Table 6:** Deviation of observed biomass from expected biomass, as a percent of observed biomass

	Unfertilized				Fertilized			
		
RemovalTreatment	B	L	M	MBL	BF	LF	MF	MBLF
Growth Form:								
Deciduous shrubs	0.7	5.4	0.5	10.7	4.0	24.6	26.0	9.6
Evergreen shrubs	5.8	2.1	3.4	12.9	–0.4	4.6	1.0	–3.0
Graminoids	7.4	4.7	2.0	–0.7	20.3	7.3	8.4	32.0
Forbs	–0.4	–0.1	0.1	–0.3	–0.4	0.3	0.01	4.7
Mosses	0.5	–0.7			0.05	0.003		
Lichens	–0.3	–5.6	1.3	–7.8	0.8	0.02	–0.2	4.6
Total community:	13.7	5.8	7.4	15.0	24.3	36.8	35.2	37.6

A positive number indicates that the observed biomass was greater than expected; a negative number indicates that observed biomass was less than expected. Expected biomass was calculated assuming that N contained in a removed species or functional type was distributed to the remaining species in proportion to their N content and biomass in the intact community. Unfertilized removal treatments were compared with the unfertilized intact community (C), while fertilized removal treatments were compared with the fertilized intact community (F).

We also compared observed and expected biomass of plants in our fertilized removal plots. Observed biomass in the fertilized removal treatments was substantially greater than expected biomass. The deviations ranged from 24% to 38% of observed values, indicating that some remaining plants were able to grow much more than expected from their performance in the fertilized intact community (Table 6). Among the different growth forms, the observed biomass of evergreen shrubs, lichens, and mosses was similar to, or slightly less than, expected in all fertilized removal treatments. In contrast, observed biomass of graminoids and deciduous shrubs was greater than expected, but the magnitude of the difference depended on whether *B. nana* had been removed. Graminoids had more biomass in fertilized treatments without *B. nana* than where it was present. For deciduous shrubs, this pattern was reversed.

## Discussion

### compensatory growth in response to removal

After 6 years of treatment, complete biomass compensation by remaining plant species had occurred in response to removal in all treatments except for MBL, which removed the most biomass. Biomass compensation is indicated by the absence of significant differences in total biomass between removal treatments and controls, and because inorganic nutrient availability returned to control levels in all unfertilized removal plots except for MBL. Removal had caused high levels of inorganic soil nutrient availability in unfertilized plots 2 years into the experiment (Bret-Harte *et al*. 2004); the much lower levels after 6 years suggest that growth of remaining species by this time was sufficient to use soil nutrients made available by the continued removal of target species. Total vascular plant production was not significantly different across any removal treatments, also suggesting that total plant growth in removal plots had recovered to control levels.

Relatively rapid biomass compensation in response to removal has been observed in alpine removal experiments (Suding *et al*. 2006). Some previous arctic removal experiments, including ours, demonstrated incomplete compensation in the short-to-medium term (Jonasson 1992; Bret-Harte *et al*. 2004), but Hobbie *et al*. (1999) inferred biomass compensation in response to removal of a single species after 4 years. Biomass in the MBL treatment will probably compensate eventually, since its vascular production is now equal to that of controls.

Contrary to our initial expectation that species in the same functional type as a removed species should respond most positively to its removal, the responding species that contributed most of the compensating biomass were from different growth forms. Removal of the dominant evergreen resulted in significantly more deciduous shrub biomass when *B. nana* was present. Removal of the dominant deciduous shrub was mostly compensated for by growth of graminoids, especially under fertilization. These results may have occurred because the remaining species in a given growth form (either deciduous shrubs or evergreen shrubs) were subordinate species that could not respond enough to offset the large response by dominant members of greater biomass in other growth forms. Our calculations based on N redistribution suggest that, as a whole, remaining plants in the different growth forms took up the N released by the removals in proportion to their biomass. While individual species differed in their N uptake capacity and growth performance, the released N was largely taken up and new biomass was produced by the remaining dominant species.

Original conceptual models of plant functional types suggested that species within a functional type, being more similar to each other in terms of nutrient use and allocation strategy, would be better at replacing each other than species from other functional types (Smith *et al*. 1997). However, our results do not support this hypothesis. It has been recognized recently that the traits that control plant *response* to perturbation often do not overlap completely with the traits that control plant *effects* on ecosystem processes (Diaz & Cabido 2001; Lavorel & Garnier 2002), because the capacity to respond to disturbance is often determined by reproductive or life history characteristics that do not affect biogeochemical cycling (Symstad 2003). The characterization of growth forms as functional types in our ecosystem is largely based on effect traits (Chapin *et al*. 1996). Where more dominant members exist within a functional type, they will likely be able to acquire more resources than their subordinates, either because they share a suite of traits permitting rapid resource acquisition, or because of their large existing biomass and extensive root systems (Díaz *et al*. 2004; Wright *et al*. 2004). Dominant and subordinate species in a given ecosystem may be similar in their contribution to a specific ecosystem function, but respond to environmental changes differently (Walker *et al*. 1999). Dominant alpine tundra species responded positively to the removal of subordinates, while subordinates responded negatively to the removal of dominants (Aksenova *et al*. 1998). Our results suggest that functional type designations based on effect traits may not be useful in predicting plant effects on ecosystem processes under changing community composition. Knowledge of the relative dominance of different plant species may be necessary for better predictions of plant response to species loss.

The physical disturbance caused by moss removal may have affected the availability of organic N for plant uptake, since attached brown moss contributes to the soil organic layer. Arctic plants rely on organic N transferred through mycorrhizal symbionts for a high proportion of their N uptake (Hobbie & Hobbie 2006), and species differ in their mycorrhizal associations and preferences for different forms of N (McKane *et al*. 2002). If different N forms were no longer available in the same ratios due to moss removal, this may have changed competitive relationships among the remaining vascular plant species. However, even when moss was not removed, compensation was due to species in growth forms other than the one removed.

The long-term outcome of species removal remains unknown. Over a longer term, successional processes may alter initial responses, but transient or subordinate species that respond to a disturbance differently than dominant species may still affect ecosystem functioning indirectly by influencing the recruitment of the dominants (Grime 1998; Symstad 2003). Thus, long-term responses to environmental change are frequently not the same as short-term responses (Shaver *et al*. 2000). Successful reproduction by seed is infrequent in our ecosystem, and our results were largely due to species that were already abundant and which likely spread vegetatively. Testing the theoretical concept of ecological ‘redundancy’ within plant functional types (Mooney 1997) would require our experiment to run until successional processes set in motion by the removal were complete, which could be a long time in tussock tundra where individual shrubs and tussocks can be well over 100 years old (Bret-Harte, unpubl. data; Mark *et al*. 1985). However, our results remain relevant to the rapid anthropogenically induced changes to climate and N cycling that are presently occurring.

### fertilization and removal

We found no increase in overall community biomass with fertilization after 6 years, despite large increases in vascular plant production. This contrasts with our earlier results (Bret-Harte *et al*. 2004), but is similar to a previous fertilization experiment in tussock tundra (Chapin *et al*. 1995). At this intermediate time, increases in the biomass of deciduous shrubs and graminoids under fertilization balanced losses in the biomass of evergreen shrubs and non-vascular plants. Species turnover and plant community reorganization were occurring under fertilization. As a result, the cycling of C and N through plant tissue was faster, but the total biomass pool remained approximately the same size.

The decline of evergreen shrub species, mosses and lichens in all the fertilized removal treatments may have been caused, at least in part, by light limitation. Light limitation due to increased vascular plant abundance and associated litter under climate warming is an important factor in the recent decline of Arctic lichen biomass (Cornelissen *et al*. 2001b). Fertilization increased litter accumulation in our experiment, especially where *B. nana* had been removed and graminoids were particularly abundant (Fig. 4a). This litter buried many low-statured evergreen species, such as *V. vitis-idaea*. However, evergreen shrubs in a range of ecosystems generally tend to decline under fertilization, as the competitive advantage conferred on ericoid mychorrhizal species (e.g. evergreen shrubs) by their ability to obtain nutrients from recalcitrant organic matter is lost in high nutrient environments (Read 1996; Cornelissen *et al*. 2001a). Our experiment demonstrated no changes in ericoid mycorrhizal colonization after 4 years of fertilization (Urcelay *et al*. 2003).

Our N redistribution calculations suggest that deciduous shrubs and graminoids are both good competitors under fertilization, but that *B. nana* is the superior competitor when it is present. *Betula nana* increasingly dominates fertilized tussock tundra in long-term experiments (Chapin *et al*. 1995; Bret-Harte *et al*. 2001; Shaver *et al*. 2001; Mack *et al*. 2004). *Betula nana* and certain graminoid species likely share some common response traits other than dominance, because they are good competitors under fertilization. For instance, both *B. nana* and *E. vaginatum* respond rapidly to fertilization by generating additional meristems that facilitate rapid new growth and may help control productivity response to favourable conditions across biomes (Bret-Harte *et al*. 2001; Knapp & Smith 2001; Bret-Harte *et al*. 2002).

### implications for ecosystem c and n cycling

The consequences of a species loss for ecosystem functioning may depend as much on the compensatory response of remaining species as it does on the direct effect of the species that is lost (Suding *et al*. 2006). In our experiment, biomass compensation led to communities with different relative abundances of plants, especially under fertilization. As seen previously, removal did not change leaf nutrient status (Fetcher 1985; Gerdol *et al*. 2002), but did lead to a redistribution of N among different community members. The shift from evergreen shrubs toward deciduous shrubs and graminoids producing more decomposable litter (Hobbie 1996; Cornelissen *et al*. 2007) should increase C and N cycling rates in these treatments and may result in lower soil C storage. As climate warms in cold biomes, changes in species composition and direct effects of temperature are expected to have a larger effect on decomposition than changes in species litter quality (Cornelissen *et al*. 2007).

Changes in species composition interact with direct effects of fertilization on plant growth and ecosystem C and N cycling. Fertilization increased the N concentration in leaves of all species in our experiment, as seen previously (e.g. Shaver & Chapin 1980; Karlsson 1985). This should positively feed back to decomposition through litter with a higher N content, although after 6 years, litter had accumulated much faster than it decomposed. Dominance by deciduous shrubs in fertilized tussock tundra over the longer term is associated with a substantial loss of soil C due to enhanced decomposition (Mack *et al*. 2004).

Climate warming is expected to increase soil nutrient availability where low temperature limits decomposition (Chapin 1983; Giblin *et al*. 1991; Nadelhoffer *et al*. 1992; Harte *et al*. 1995). Many natural ecosystems are also now impacted by deposition of anthropogenically-fixed N (Vitousek *et al*. 1997). Biodiversity loss due to N deposition has already been seen in temperate ecosystems (Stevens *et al*. 2004), and N deposition is expected to continue increasing over the next 50 years (Galloway *et al*. 2004). Analysis of N-addition experiments across a range of North American ecosystems suggests that cold regions with soils of low cation exchange capacity, including much of the Arctic, may be particularly vulnerable to species loss with N deposition (Clark *et al*. 2007).

Our experiment suggests that, at least on a decadal time scale, arctic tundra ecosystems in a more fertile and warmer future may be dominated either by deciduous shrubs or by graminoids. Which growth form dominates will likely depend on its current local abundance and the prevailing disturbance regime. Herbivory is one important factor controlling transitions between dominance by grasses and shrubs in savannas (Folke *et al*. 2004). Widespread deciduous shrub expansion has recently been observed in both arctic and alpine ecosystems (Sturm *et al*. 2001; Tape *et al*. 2006; Cannone *et al*. 2007). Deciduous shrubs, often dominated by *B. nana*, are common in moist acidic tundra on mesic slopes of older landscapes (Walker *et al*. 1994; Walker *et al*. 1995). However, vast areas of non-acidic tundra have much lower relative abundance of deciduous shrubs, with only rare occurrences of *B. nana* (Walker *et al*. 1994; Walker *et al*. 1995; Hobbie *et al*. 2005). Notably, fertilization has led to dominance by either deciduous shrubs or graminoids in different experiments in the European and North American Arctic (reviewed by Dormann & Woodin 2002; van Wijk *et al*. 2003). Moreover, once graminoids have become dominant due to fertilization, recovery appears to be very slow (Brancaleoni & Gerdol 2006). Pollen records suggest that both herb-graminoid tundra (or steppe) and shrub tundra dominated by *B. nana* or *Betula glandulosa* have been widespread and stable vegetation types at different times between 18 000 and 6000 years ago in northern Alaska (Anderson & Brubaker 1994). Our results suggest that present plant distributions may lead to two different stable-states, graminoid-dominated steppe and shrub tundra, as climate warming continues in the Arctic.

## Supplementary material

The following supplementary material is available for this article:

Appendix S1 Equations for calculation of expected biomass in removal treatments.

Figure S1 Biomass removed in each year of the experiment.

Figure S2 Depth of thaw in 2003.

This material is available as part of the online article from: http://www.blackwell-synergy.com/doi/abs/10.1111/j.1365-2745.2008.01378.x (This link will take you to the article abstract).
