# Supplementary material for: Plant functional types do not predict biomass responses to removal and fertilization in Alaskan tussock tundra
Source: J Ecol. 2008 Jul;96(4):713–26. doi: 10.1111/j.1365-2745.2008.01378.x (PMC2438444; doi:10.1111/j.1365-2745.2008.01378.x)
Supplement: Appendix S1 — Equations for calculation of expected biomass in removal treatments. [file jec0096-0713-SD1.doc]

**Appendix S1**. Equations for the calculation of expected biomass of remaining species in removal treatments.

To explore the behavior of remaining species after the removal of selected species from the fertilized and unfertilized neighbor removal treatments, we calculated the expected biomass of species if the amount of nitrogen (N) contained in the removed species were reallocated among the remaining species in proportion to their N content in the no removal treatments (control or fertilized). In each experimental treatment we measured biomass$B_{ij}$ and N concentration $\%N_{ij}$ for each species *j* and tissue *i*. From these two we calculated:

(1) $N_{ij}=\left(\%N_{ij}\times B_{ij}\right)/100$,

(2) $N_{j}=\sum_{i} N_{ij}$,

(3) $N_{T}=\sum_{j} N_{j}$,

(4) $\alpha_{ij}=\frac{N_{ij}}{N_{j}}$,

(5) $\gamma_{j}=\frac{N_{j}}{N_{T}}$.

We assume that $\%N_{ij}$ and $\alpha_{ij}$, the relative allocation of N to different tissue types within a species, both remained unchanged. Thus, if N mass was added to a species in a removal treatment, its biomass increased in proportion to its N content in the no removal treatment.

The mass of nitrogen contained in the removed species was calculated as:

(6) $N_{\text{removed}}=\sum N_{k}$, for all removed species, *k*.

This amount, *N*_removed_, of nitrogen was reallocated among the remaining species in proportion to their N content in the no removal treatment, so that the total amount of nitrogen in aboveground plant biomass in the treatment remained constant:

(7) $\gamma_{j}^{*}=\gamma_{j}+\gamma_{j}\left(\frac{\sum_{k} \gamma_{k}}{1-\sum_{k} \gamma_{k}}\right)$,

where $\gamma_{k}$ represents the original fraction of total nitrogen in aboveground biomass that was found within each removed species. The tissues of each remaining species received $\gamma_{j}^{*}$ fraction of *N*_removed_ :

(8) $N_{ij}^{*}=N_{ij}+\alpha_{ij}\gamma_{j}^{*}N_{\text{removed}}$.

To calculate the amount of total biomass in each of the remaining species in a removal treatment, from equation (1) we have

(9) $\begin{matrix} B_{ij}^{*}=\frac{100N_{ij}^{*}}{\%N_{ij}} \\ B_{j}^{*}=\sum_{i} B_{ij}^{*} \end{matrix}$.

**Symbol Definitions**

$\alpha_{ij}$ proportion of nitrogen in species *j* found within tissue type *i*

$B_{ij}$ biomass of species *j*, tissue *i* before nitrogen redistribution

$B_{ij}^{*}$ biomass of species *j*, tissue *i* after nitrogen redistribution

$B_{j}^{*}$ biomass of species *j* after nitrogen redistribution

$\gamma_{j}$ proportion of total plot nitrogen found within species *j* before nitrogen redistribution

$\gamma_{j}^{*}$ proportion of total plot nitrogen found within species *j* after nitrogen redistribution

$\%N_{ij}$ percent nitrogen in species *j*, tissue *i*

$N_{ij}$ mass of nitrogen in species *j*, tissue *i* before nitrogen redistribution

$N_{ij}^{*}$ mass of nitrogen in species *j*, tissue *i* after nitrogen redistribution

$N_{j}$ mass of nitrogen in species *j* before nitrogen redistribution

$N_{\text{removed}}$ mass of nitrogen in all removed species

$N_{T}$ total mass of nitrogen in a plot
