# Supplementary material for: Plant functional types do not predict biomass responses to removal and fertilization in Alaskan tussock tundra
Source: J Ecol. 2008 Jul;96(4):713–26. doi: 10.1111/j.1365-2745.2008.01378.x (PMC2438444; doi:10.1111/j.1365-2745.2008.01378.x)
Supplement: Figure S2 — Depth of thaw in 2003. [file jec0096-0713-SD3.doc]

**Figure S2.** Depth of thaw in early September, 2003. Removal treatment abbreviations: B = removal of *Betula nana*, L = removal of *Ledum palustre*, M = removal of all mosses, MBL = combined removal of *Betula nana*, *Ledum palustre*, and all mosses. Error bars indicate 1 SE among blocks (n=6).
